# Progress in Disease of Digestive Organs

**Published:** 1903-06-20

**Authors:** 


*(To be continued.)*

### Progress in Disease of Digestive Organs.

*(Continued from page* 188.)

**Gastric Ulcer.**—Walko[7] finds that olive oil is useful in this disease when hyperacidity is present. It inhibits the secretion of H.Cl. and coats the ulcer with a non-irritating film which prevents pain. It does not interfere with the motility of the stomach, and it relieves the constipation when given in tablespoonful doses. If the patient objects to it, an emulsion may be introduced by the stomach tube. No other food was given in acute cases with hæmatemesis for several days. Striking results were also obtained in chronic cases, even of the most obstinate forms. Kelling[8] argues that the tenderness in the epigastrium and to the left of the twelfth dorsal vertebra is not due to the nearness of the ulcer, but to hyperæsthesia of the sympathetic. This nerve condition leads to pyloric spasm and retards healing. He advises full doses of bromide and codeia by the rectum with or without atropine, and reports that he has had excellent results by this method of treatment. The absence of symptoms in undoubted ulcers is often remarkable. Thus the case of a latent ulcer in a woman aged 57 is reported by Cooper Ashurst[9] which perforated into the lesser peritoneal cavity with few symptoms. A second perforation also in the lesser curvature from the large chronic ulcer led to a second abscess which finally broke into the general peritoneum, causing death before any definite indication of an ulcer appeared. F. J. Smith[10] believes that most ulcers originate in minute erosions or perforations due to suppuration of the adenoid tissue beneath the mucosa. These erosions are a frequent source of hæmatemesis, and may cause few or no prior symptoms. Localised, as opposed to general tenderness, would point to ulcer rather than erosion. We must also diagnose them from the effects of a movable kidney, acute gastritis, sick headaches, and acid dyspepsia. In treating ulcers with marked dyspeptic symptoms in anæmic girls unless the tongue is badly furred, iron does good at once without a preparation by bismuth. Rest in bed causes a quick improvement, and in a few days if there is no ulcer, or if it is healed, a test draught of salt ʒi water ℥ii will cause no smarting. Raw milk should not be given without some addition to prevent large clots forming. In other forms of ulcer, if bleeding occurs, say, three times in a week, or if in chronic cases no benefit comes from rest and bleeding or dilatation of the stomach occurs, an operation should be offered. In the discussion at the Medical Society, B. Dawson[11] spoke of hæmatemesis from capillary oozing and varicose æsophageal veins, bleeding due to menstruation, even when there are existing ulcers, or due to nerve states after operations, or to excretion of some poisons, and even during urticaria. Hale White referred to the numerous cases of capillary oozing in young women which continued for years with intervals of good health and little wasting. Rolleston mentioned the bleeding in splenic anæmia, dysentery, and in rare cases of typhoid, renal disease, ulcerating growths, toxic erosions, as in pneumonia, and in rare instances in heart failure. Erosions are not, he thinks, due to changes in lymphoid follicles, nor are they early stages of gastric ulcers. Among the remedies referred to were adrenalin and gelatine given by the mouth, perchloride of iron, calcium chloride and silver nitrate, cessation from all food, and surgical intervention in repeated attacks, especially if the symptoms point to actual ulcers. Max Einhorn[12] advises rest and starvation, opiates, ice, and hypodermics of adrenalin or gelatine.

**Tumours.**—Nuthall and Emanuel [13] describe three remarkable cases of diffuse carcinomatosis, affecting both the stomach the small and large intestines. The stomachs were of the "leather bottle" type, and the intestines were infiltrated from the attached border for a varying distance towards the opposite side. The growth was found to be a glandular carcinoma undergoing colloid degeneration, and spreading in the submucosa and subserous layers. To the naked eye the appearance was that of diffuse fibrosis, so that, when first shown, it was suggested that the growths were endotheliomata. Among the symptoms were gastric and intestinal colic, obstruction, general emaciation, and asthenia. Einhorn [14] diagnosed the syphilitic nature of a gastric tumour, and the correctness of his view was proved by its disappearance under specific remedies. There was a history of syphilis, and of gastric troubles lasting seven years. Hydrochloric acid was present, and there was but little loss of weight. With regard to the origin of cancer from old standing ulcers, Borrmann [15] could not find in 63 cases a single one in which this mode of origin could be proved. Krokiewicz says, too, that he had not found an instance in 7,000 post-mortems. On the other hand, Bockelman, in Holland, declares that half the cases of cancer of the stomach originate in this way, and that such cases show much less interstitial change and less atrophy of the glands. Hence the secretory activity is retained longer than in primary cancer.

**Gastroptosis.** — A remarkable analysis of the anatomy of enteroptosis is given in Keith's Hunterian Lectures.[16] He points out that the position of the viscera is due to the form of respiratory movements, and that with a deviation of the action of the muscles of respiration their position is altered. Thus the viscera are poised between the muscles of expiration and inspiration, and when the inspiatory muscles overbalance the others a fall of the viscera or enteroptosis takes place. The domes of the diaphragm instead of lying behind the fifth costal cartilages in the cadaver, *i.e.* in complete expiration, were found in 50 bodies to vary between the third and the sixth, a range of more than $3\frac{1}{2}$ inches. Displacement of the stomach and liver is due either to paresis of abdominal muscles, or else the body cavity is so constricted by clothing or disease that these organs cannot swing forwards and override each other. Francine,[17] in a study of 100 cases, refers to the exciting causes as failure of abdominal muscles from pregnancies, etc., chlorosis, dyspepsia, corsets, spinal curvature, uterine displacements, enlargements of the liver, and traumatism. Enteroptosis exists most often in multiparous women, but is found at all ages even in children. To detect it the best plan is to inflate the stomach with air through a tube. Serious nerve symptoms and motor failure of the stomach are common sequelæ of the disease. Lavage diet, massage, and exercises for the abdominal muscles are indicated, and occasionally operative help. A. W. W. Lea [18] recommends Swedish gymnastic exercises performed six to twelve times night and morning. Thus the patient raises herself from the level to a sitting posture without using her arms, and again lying down raises each leg vertically, and finally after emptying the lungs closes the glottis and then makes a deep inspiration. An abdominal support, or a straight-fronted corset, may be of use. Lichty [19] lays stress on the chronic enteritis which often accompanies this condition.

**Dilatation.**—Clifford Allbutt [20] speaks of the importance in treatment of rest before and after meals, which should be small in bulk and frequent, and accompanied with as little fluid as possible. Bismuth, bromides, rhubarb, strychnia, and, if there is hyperacidity, carbonate of magnesia may be needed. Bardswell notices that in those consumptives who are kept in bed for months from the severity of their attack there is a risk of dilatation from the large amount of food given them. With early cases where rest in bed is not necessary there is but little tendency to this complication under sanatorium treatment. Broadbent refers to the anginal attacks, vertigo, sleeplessness, and palpitation which dilatation of the stomach is apt to cause, and adds that it may even bring on a fatal issue in cases of failing heart. Tetany and Hodgkin's disease are sometimes found with it. Savill recommends in some cases treatment by the Salisbury diet and the application of the galvanic current. Saundby [21] has found that a draught containing 2 drachms of bicarbonate of soda followed by one of 90 grains of tartaric acid, if given on an empty stomach, is a reliable, safe, and easy test. Its use has enabled him to affirm that atony and dilatation is much more common than is usually thought. It is caused by neurasthenia or any debilitating illness, and the prognosis does not depend on the extent of dilatation. In about half the cases the stomach reaches to the level of the anterior superior spines. He puts severe cases in bed on one ounce of milk diluted with barley water every hour till pain ceases. This is gradually increased, and four ounces of minced chicken is next added twice daily. If milk fails, lavage or a diet of minced meat alone may be tried. Simple tonics with bismuth or acid may be given.

[7] Brit. Med. Jour., Jan. 24. [8] Brit. Med. Jour., Jan. 31. [9] Amer. Jour. Med. Sci., Oct. [10] Clin. Jour., Nov. 19. [11] Brit. Med. Jour., Nov. 29. [12] Med. Rec., March 7. [13] Lancet, Jan. 17. [14] Brit. Med. Jour., Feb. 28. [15] Bristol Med. Chir. Jour., Dec. [16] Lancet, March 7. [17] Med. Rec., Feb. 14. [18] Med. Chron., p. 235, 1902. [19] Med. Rec., Nov. 29. [20] Brit. Med. Jour., Nov. 1. [21] Brit. Med. Jour., Nov. 29.

*(To be continued.)*

